# Up-regulation of matrix metalloproteinases in a mouse model of chemically induced colitis-associated cancer: the role of microRNAs

**DOI:** 10.18632/oncotarget.3027

**Published:** 2015-01-23

**Authors:** Feiyan Ai, Xuemei Zhang, Xiayu Li, Zailong Qin, Qiurong Ye, Li Tian, Anliu Tang, Nan Li, Guiyuan Li, Jian Ma, Shourong Shen

**Affiliations:** ^1^ Department of Gastroenterology, The Third Xiangya Hospital of Central South University, Changsha, Hunan, China; ^2^ Hunan Key Laboratory of Nonresolving Inflammation and Cancer, Changsha, Hunan, China; ^3^ Hunan Cancer Hospital and the Affiliated Cancer Hospital of Xiangya School of Medicine, Cancer Research Institute, Central South University, Key Laboratory of Carcinogenesis, Ministry of Health, Key Laboratory of Carcinogenesis and Cancer Invasion, Ministry of Education, Changsha, Hunan, China

**Keywords:** colitis-associated cancer, Dicer1, matrix metalloproteinases, microRNAs, non-resolving inflammation

## Abstract

Emerging evidence has implicated microRNAs in regulating the production of multiple inflammatory mediators including cytokines and chemokines. We previously elucidated the dynamic activation of key signals that link colitis to colorectal cancer. In this study, we observed a sharp increase in the levels of matrix metalloproteinases (Mmps) that provided a basis for the inflammation-cancer link, and we questioned whether this was a consequence of the dysregulation of Mmp-specific microRNAs, at least partly. We assayed a panel of murine microRNAs that were predicted to target Mmps and found they were downregulated in the inflammation-cancer link. Furthermore, we demonstrated that three murine microRNAs, namely miR-128, -134, and -330, can target the three Mmps Mmp3, Mmp10, and Mmp13, respectively. We also found that the level of the microRNA-processing enzyme Dicer1 was decreased in the inflammation-cancer link. These microRNAs functioned as tumor suppressors in colon cancer cells, attenuating the proliferation, migration, and invasion potential of murine colon cancer cells as well as angiogenesis and the growth of tumors derived from these cells. Our results suggest that microRNAs modulate the production of key inflammatory mediators and that microRNA dysfunction may contribute to the non-resolving inflammation associated with cancer.

## INTRODUCTION

Inflammation tends to develop when an organism is exposed to a harmful physical or chemical agent(s), infection, or an autoimmune disease. Approximately one and half centuries ago, when the German pathologist Rudolph Virchow first observed the infiltration of leukocytes in tumor tissues, the correlation between inflammation and cancer caught the public's attention. By 2011, inflammation had become viewed as a hallmark of cancer [1]. Indeed, inflammation, particularly chronic inflammation, plays an important part in the development and progression of many solid tumors, for example, gallbladder cancer [2], gastric cancer [3], prostate cancer [4], and colorectal cancer [5]. Therefore, disruption of the link between chronic inflammation and cancer could have profound and far-reaching positive consequences.

Ulcerative colitis is an immune-mediated inflammatory disease in which acute and chronic inflammation coexist over long periods. Patients with ulcerative colitis are at increased risk for developing colorectal cancer, namely colitis-associated cancer (CAC). CAC typifies the inflammation–cancer link, including inflammation, dysplasia, and ultimately carcinoma. In a mouse model, a single pretreatment with azoxymethane (AOM) with subsequent repeated ingestion of dextran sodium sulfate (DSS) leads to colon cancer in ~100% of mice [6]. This AOM/DSS-induced CAC mouse model simulates the histological changes of the colon observed in CAC patients. With this model, we discovered that different key signaling pathways [NF-κB, STAT3, p38 mitogen-activated protein kinase (MAPK), and Wnt/β-catenin] and their target genes are hyperactivated in different phases of inflammation-associated cancer [7]. Among the genes activated by these signaling pathways, the dramatic upregulation of those genes encoding matrix metalloproteinases (Mmps) attracted our attention.

The Mmp family is comprised of structurally related, zinc-dependent endopeptidases [8]. They can degrade the extracellular matrix and thus contribute to the invasive and metastatic potential of tumors [9]. Mmps also play a critical role in various inflammatory, infectious, and repair processes, activating signal transduction pathways that control cytokine biosynthesis and precisely cleaving most of the chemotactic proteins involved in inflammatory cell and immune cell recruitment [10, 11]. The levels of transcription, zymogen activation, and endogenous inhibition controlling the proteolytic activity and expression of Mmps have been studied intensively [9].

Recent evidence strongly suggests that microRNAs (miRNAs) play crucial roles in inflammation. For example, miR-155 is upregulated and miR-125b is downregulated in lipopolysaccharide (LPS)-stimulated mouse RAW264.7 macrophages, and these miRNAs participate in regulating the response to endotoxin shock by influencing the transcription and translation of TNF-α [12]. MiR-98-mediated posttranscriptional control is involved in fine tuning interleukin (IL)-10 production in endotoxin tolerance [13]. The role of miRNAs in inflammatory diseases of the lung and skin and in endothelial cell inflammation and rheumatoid arthritis also has been reported [14]. Therefore, we used a mouse model to explore whether the upregulation of Mmps in inflammation-cancer link is partly due to dysregulation of miRNAs.

## RESULTS

### *Mmp3*, *Mmp10*, and *Mmp13* are upregulated during murine CAC progression

**Figure 1:**
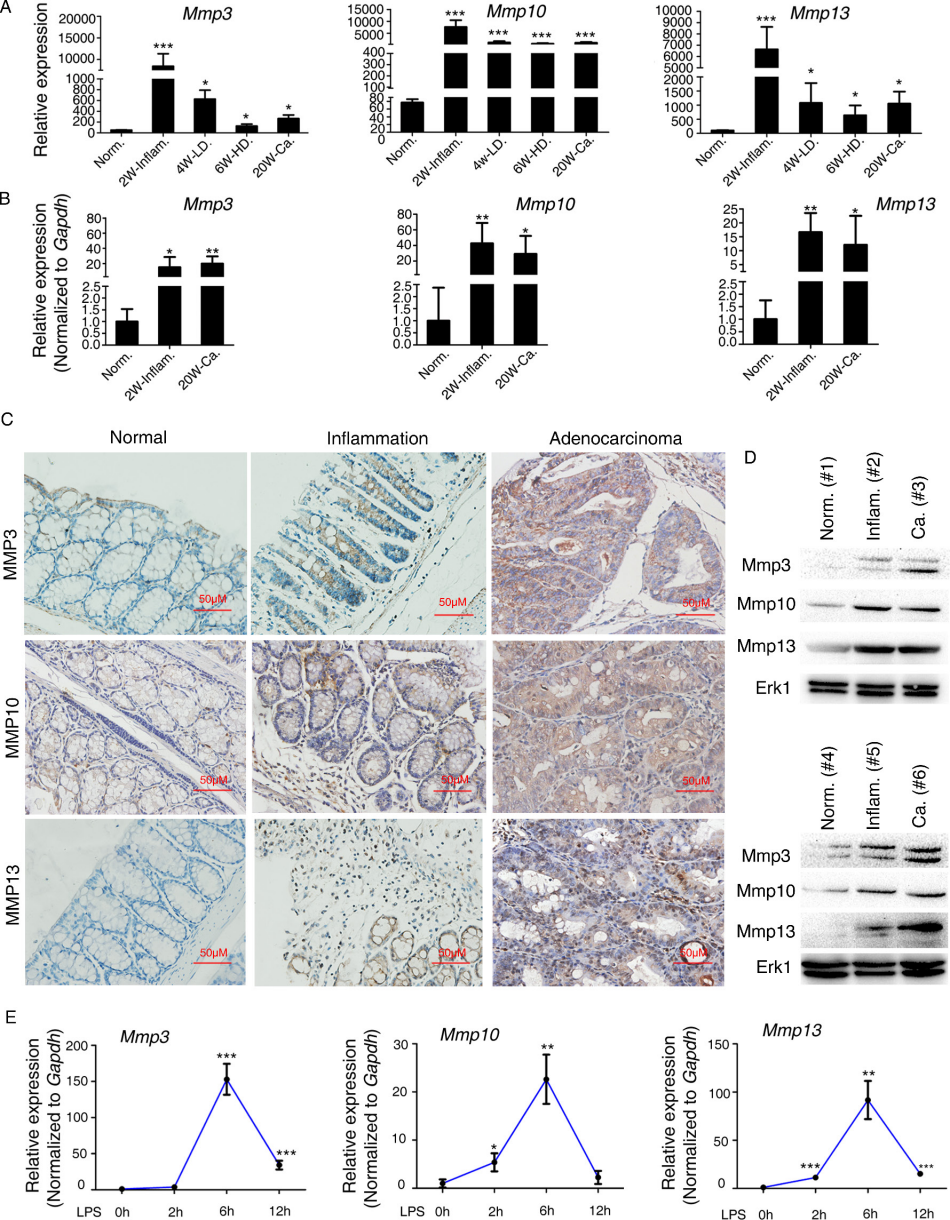
*Mmp3*, *Mmp10*, and *Mmp13* are upregulated during CAC progression in mice **(A)** Recapitulated are the mRNA expression patterns of *Mmp3*, *Mmp10*, and *Mmp13* based on our previous microarray analysis (GSE31106**[; ref. 7]**) of the AOM/DSS-induced CAC mouse model. Abbreviations: Norm, normal; Inflam, inflammation; LD, low-grade dysplasia; HD, high-grade dysplasia; Ca, adenocarcinoma. **(B)** qRT-PCR analysis of the mRNA levels of *Mmp3*, *Mmp10*, and *Mmp13* in mouse colitis tissue, CAC tissue, and normal colon tissue. Relative expression of *Mmp3*, *Mmp10*, and *Mmp13* was determined by using the 2^−ΔΔCt^ method. **(C)** Representative expression patterns of Mmp3, Mmp10, and Mmp13 (determined by immunohistochemistry) in mouse colitis tissue, CAC tissue, and normal colon tissue. **(D)** Representative expression patterns of Mmp3, Mmp10, and Mmp13 (determined by western blot) in mouse normal colon tissue (Norm.) colitis tissue (Inflam.) and CAC tissue (Ca.). **(E)** Kinetics of LPS-induced *Mmp3*, *Mmp10*, and *Mmp13* expression in RAW264.7 murine macrophages. RAW264.7 cells were stimulated with LPS (100 ng/ml) for 0, 2, 6, or 12 h. *Mmp3*, *Mmp10*, and *Mmp13* mRNAs were quantified by qRT-PCR. All data are shown as the mean ± SD. *p < 0.05, **p < 0.01, ***p < 0.001 compared with control.

The AOM/DSS-inducible CAC mouse model is a genetically stable and clinically relevant animal model of CAC that closely mimics the pathological course of human ulcerative colitis developing to colorectal tumors [15]. By employing this model, we previously reported comprehensive gene expression data for murine CAC tissues (acquired with the Affymetrix mouse 430 2.0 Genome Array), including inflamed lesions, and assigned a pathological grade to each tissue [7]. Here we confirmed those findings, and we observed a dramatic increase in *Mmp3*, *Mmp10*, and *Mmp13* expression in CAC tissue relative to normal tissue (Figure 1A); their expression was increased in dysplastic tissues and adenocarcinoma tissues and particularly in inflamed tissues. Using the same mouse model, we further assessed the mRNA expression levels of *Mmp3*, *Mmp10*, and *Mmp13* using quantitative real-time PCR (qPCR) of samples of mouse colitis tissue as well as CAC and normal colon tissues (Figure 1B). These mRNAs were upregulated, consistent with our previous Genome Array results [7]. Moreover, the levels of Mmp3, Mmp10, and Mmp13 proteins were also upregulated as determined with immunohistochemistry and western blotting of mouse colitis tissues and CAC tissues compared with normal colon tissues (Figure 1C,1D). To further generalize our findings, we used LPS to stimulate murine macrophages (RAW264.7) to imitate inflammation and measured *Mmp3*, *Mmp10*, and *Mmp13* expression over time; the mRNA levels were significantly increased (Figure 1E), indicating that these three genes are upregulated in inflammation-cancer link. Furthermore, we recapitulated the gene expression levels of *Mmp3, Mmp10,* and *Mmp13* from large cohorts of ulcerative colitis (UC) and colorectal cancer (CRC) patients that are available from the GEO database (GSE38713 and GSE37364). As shown in Supplementary Figure 1A, *Mmp3, Mmp10,* and *Mmp13* mRNA levels were significantly upregulated in ulcerative colitis and colorectal adenocarcinoma compared with the levels of normal cohorts. We also used qRT-PCR to assay their levels in human CRC tissues. The overall average mRNA expression levels of *Mmp3, Mmp10,* and *Mmp13* were higher in tumor than that in normal tissues (Supplementary Figure 1B, left). A variety of factors could explain our observed increase in Mmps expression, such as upregulated expression of activators of transcription, including AP-1, PEA3, and STAT, and hypomethylation of CpG sites and hyperacetylation of the *Mmp* promoters [11, 16]. A recent study reported the widespread involvement of miRNAs in regulating inflammation, and thus we were curious about whether the upregulation of Mmps during CAC progression is partly influenced by miRNAs.

### Murine miR-128, miR-134, and miR-330 directly target and inhibit *Mmp3*, *Mmp10*, and *Mmp13*, respectively

**Figure 2:**
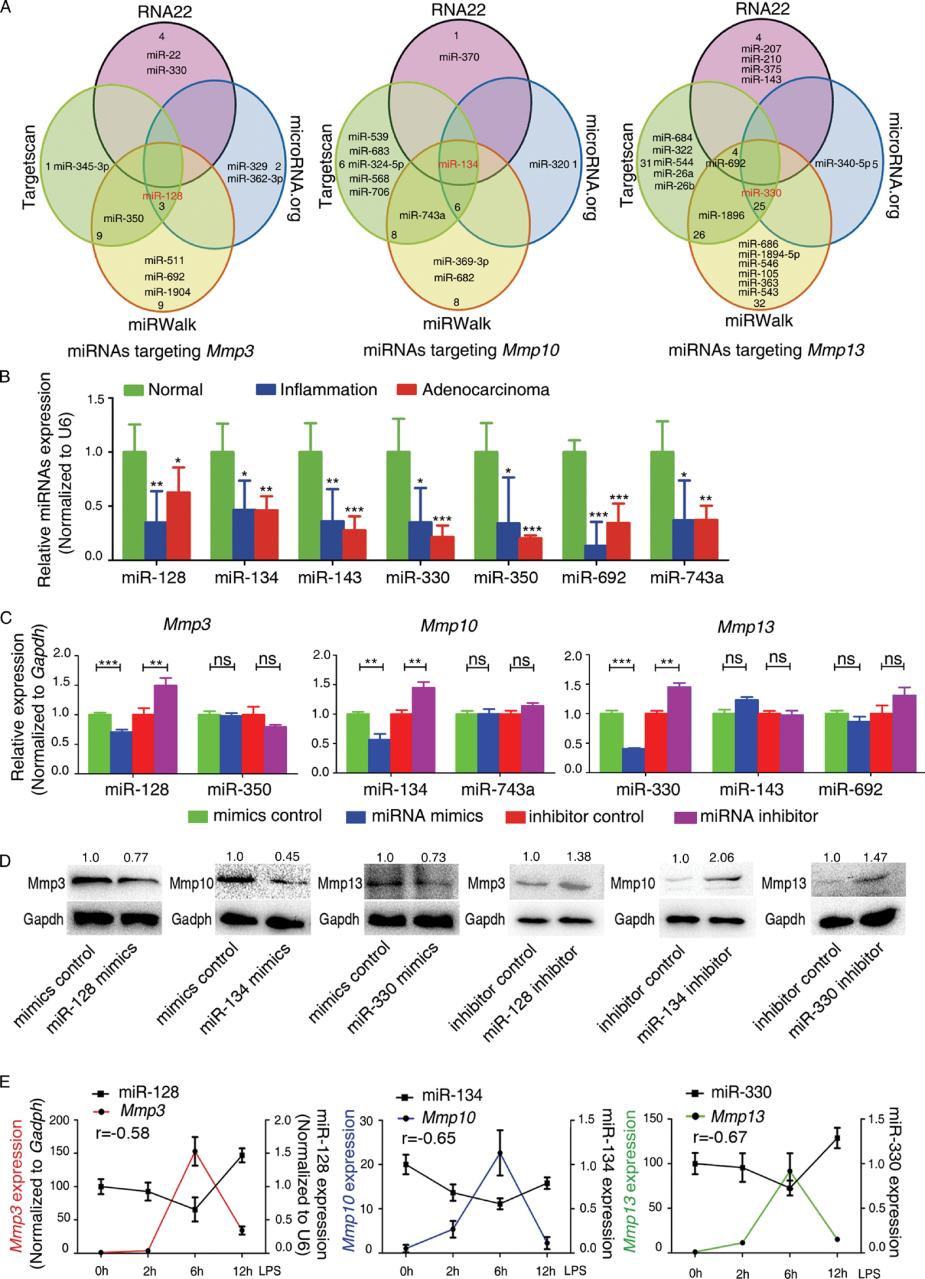
Inflammation-dependent downregulation of miR-128, miR-134, and miR-330 during murine CAC progression and an inverse correlation with the levels of Mmp3, Mmp10, and Mmp13 **(A)** Venn diagram displaying the prediction of murine miRNAs targeting *Mmp3*, *Mmp10*, and *Mmp13* mRNAs, respectively. **(B)** qRT-PCR was performed to confirm the expression of the selected miRNAs in mouse colitis tissue, CAC tissue, and normal colon tissue. Several miRNAs including the seven shown here were downregulated in mouse colitis tissue and CAC tissue compared with normal colon tissue. Relative expression of each miRNA was determined with the 2^−ΔΔCt^ method. U6 was used as the internal control, and the expression values for “normal” tissue were set as 1. **(C)** MiRNA mimics or inhibitors were transfected into CT26.WT cells, and the expression of *Mmps* was determined by qRT-PCR. *Gapdh* was used as the internal control, and the expression values of “mimics or inhibitor control” were set as 1. **(D)** Transfection with miR-128, miR-134, or miR-330 mimic decreased Mmp3, Mmp10, and Mmp13 protein levels, respectively, whereas transfection with the miR-128, miR-134, or miR-330 inhibitor increased Mmp3, Mmp10, and Mmp13 levels, respectively, in CT26.WT cells. **(E)** Relative expression of *Mmp3*, *Mmp10*, and *Mmp13* and of miR-128, miR-134, and miR-330 in LPS-stimulated murine macrophages (RAW264.7) revealed a negative correlation based on Spearman analysis (r = –0.58, *p* < 0.01; r = −0.65, *p* < 0.001; r = −0.67, *p* < 0.001). All data are shown as the mean ± SD. *p < 0.05, **p < 0.01, ***p < 0.001 compared with control.

To predict whether miRNAs target *Mmp3*, *Mmp10*, and/or *Mmp13* in murine colon cancer cells, we first utilized the bioinformatics algorithms TargetScan, miRWalk, microRNA.org, and RNA22. We identified seven murine miRNAs (miR-128, 134, 143, 330, 350, 692, 743a) that were predicted to target *Mmp* mRNAs by at least two of the four algorithms (Figure 2A). We then analyzed the levels of these seven miRNAs in mouse colitis tissue, CAC tissue, and normal colon tissue using reverse transcription (RT)-coupled PCR. Indeed all seven miRNAs were dramatically decreased in mouse colitis tissues and CAC tissues compared with normal colon tissues (Figure 2B). Furthermore, there was an opposite expression trend between the seven miRNAs and *Mmp3*, *Mmp10*, and *Mmp13* (Figure 1B and 2B).

We then screened for the effects of the seven predicted miRNAs on *Mmp3*, *Mmp10*, and *Mmp13* expression using the following methods. First, to test whether these miRNAs could inhibit expression of *Mmp3*, *Mmp10*, and *Mmp13*, we transfected each of the seven miRNA mimics (or miRNA inhibitors) into the murine colon cancer cell line CT26.WT and found that the miR-128, miR-134, and miR-330 mimics reduced the levels of *Mmp3*, *Mmp10*, and *Mmp13* mRNAs, respectively, whereas each of the inhibitors increased *Mmp3*, *Mmp10*, and *Mmp13* levels, respectively (Figure 2C). No significant correlations were found between the miR-350 mimic or inhibitor and the level of *Mmp3*, and likewise for miR-743a and *Mmp10* and for miR-143/miR-692 and *Mmp13* (Figure 2C). Western blotting revealed that miR-128, miR-134, and miR-330 decreased the levels of Mmp3, Mmp10, and Mmp13, respectively (Figure 2D). Furthermore, the expression levels of miR-128, miR-134, and miR-330 correlated negatively with those of *Mmp3*, *Mmp10*, and *Mmp13*, respectively, in a macrophage model of inflammation (r = –0.578, r = –0.65, r = –0.668, respectively; Figure 2E). These results suggested that murine miR-128, miR-134, and miR-330 target *Mmp3*, *Mmp10*, and *Mmp13*, respectively. In addition, we assayed the expression levels of miR-128, miR-134, and miR-330 in human colorectal cancer and normal colonic tissues. When compared with normal colonic tissues, there was a significantly decreased expression of miR-128, miR-134, and miR-330 detected in the colorectal cancer specimens (Supplemental Figure 1B, right).

**Figure 3:**
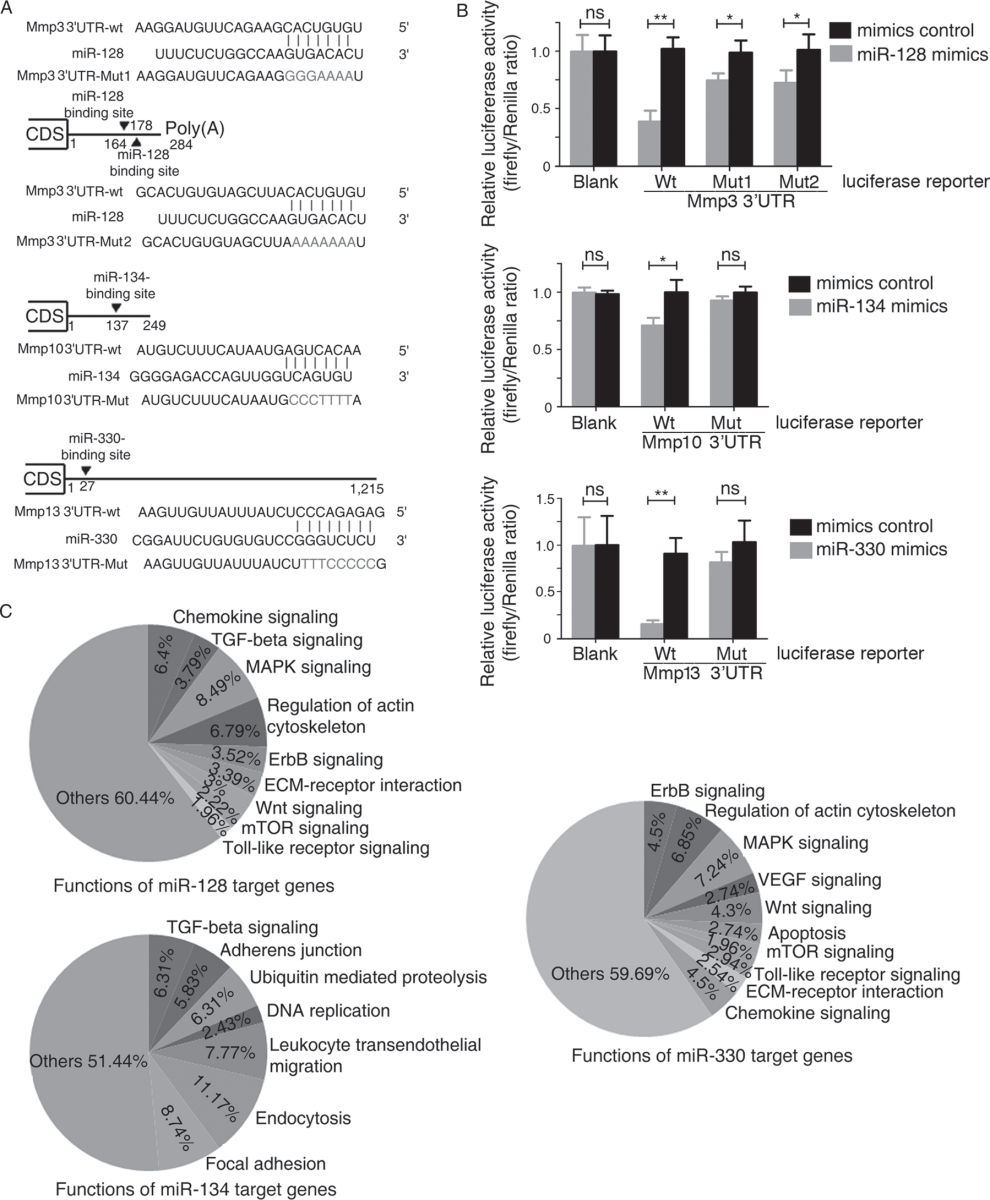
*Mmp3*, *Mmp10*, and *Mmp13* are direct targets of murine miR-128, miR-134, and miR-330, respectively **(A)** Scheme for the potential binding site of miR-128, miR-134, and miR-330 in the 3′-UTR of *Mmp3*, *Mmp10*, and *Mmp13* and the sequence of each intact miR-128, miR-134, and miR-330 binding site (wild-type, wt) and its mutant (Mut) within the luciferase reporter vector. **(B)** Luciferase assay with HEK293 cells, which were cotransfected with the indicated miRNA mimics (or control) and a luciferase reporter containing the 3′-UTR (wild type or mutant) of the indicated Mmps. An empty luciferase reporter construct was used as a negative control (Blank). Luciferase activities were measured 36 h post-transfection. miR-128, miR-134, and miR-330 suppressed the luciferase activity of *Mmp3*, *Mmp10*, and *Mmp13*, respectively, in luciferase wild-type reporter constructs. The data are the mean ± SD for separate transfections (n = 3). *p < 0.05, **p < 0.01 compared with control. **(C)** Based on an analysis with the DAVID 2008 Functional Annotation Bioinformatics Microarray Analysis Tool, the pie chart shows the percentage of predicted target genes that may be involved in different pathways.

To verify whether *Mmp3*, *Mmp10*, and *Mmp13* are direct targets of miR-128, miR-134, and miR-330, respectively, we used a luciferase assay to test the binding of each miRNA to the respective gene's 3′ untranslated region (UTR). As shown in Figure 3A, two miR-128-binding sites were identified in the 3′-UTR of *Mmp3* mRNA, and likewise one miR-134-binding site was identified for *Mmp10* mRNA and one miR-330-binding site was identified for *Mmp13* mRNA. There was perfect base pairing between the seed sequence of mature miR-128/miR-134/miR-330 and the 3′-UTR of *Mmp3/Mmp10/Mmp13* mRNAs, respectively. We subcloned the full-length *Mmp3* 3′-UTR, *Mmp10* 3′-UTR, and *Mmp13* 3′-UTR into a luciferase reporter vector. Figure 3B shows that addition of *in vitro* transcribed miR-128, miR-134, and miR-330 mimics significantly suppressed the luciferase activity of the *Mmp3*, *Mmp10*, and *Mmp13* 3′-UTR upon cotransfection with the luciferase vector (wild-type, mutant, or blank control) with the *in vitro* transcribed miRNA (miR-128, miR-134, miR-330, or control) mimics into human embryonic kidney (HEK293) cells. These results provided evidence that each of miR-128, miR-134, and miR-330 directly recognizes the respective 3′-UTR of the *Mmp3*, *Mmp10*, and *Mmp13* mRNAs and thereby inhibits their translation.

We next searched for possible target genes of miR-128, miR-134, and miR-330 using web-based bioinformatics algorithms. A total of 3761, 1020, and 1686 possible targets for miR-128, miR-134, and miR-330, respectively, were predicted by TargetScan, miRWalk, and miRanda. We then used DAVID Bioinformatics Resources Analysis Tools to classify the function of these target genes, revealing that most target genes of miR-128, miR-134, and miR-330 are involved in signaling pathways such as MAPK, Wnt, TGF-β, and mTOR and in the function of adherens junctions (Figure 3C), all of which are important for tumorigenesis.

### The microRNA-processing enzyme Dicer1 is decreased in the inflammation-cancer link

**Figure 4:**
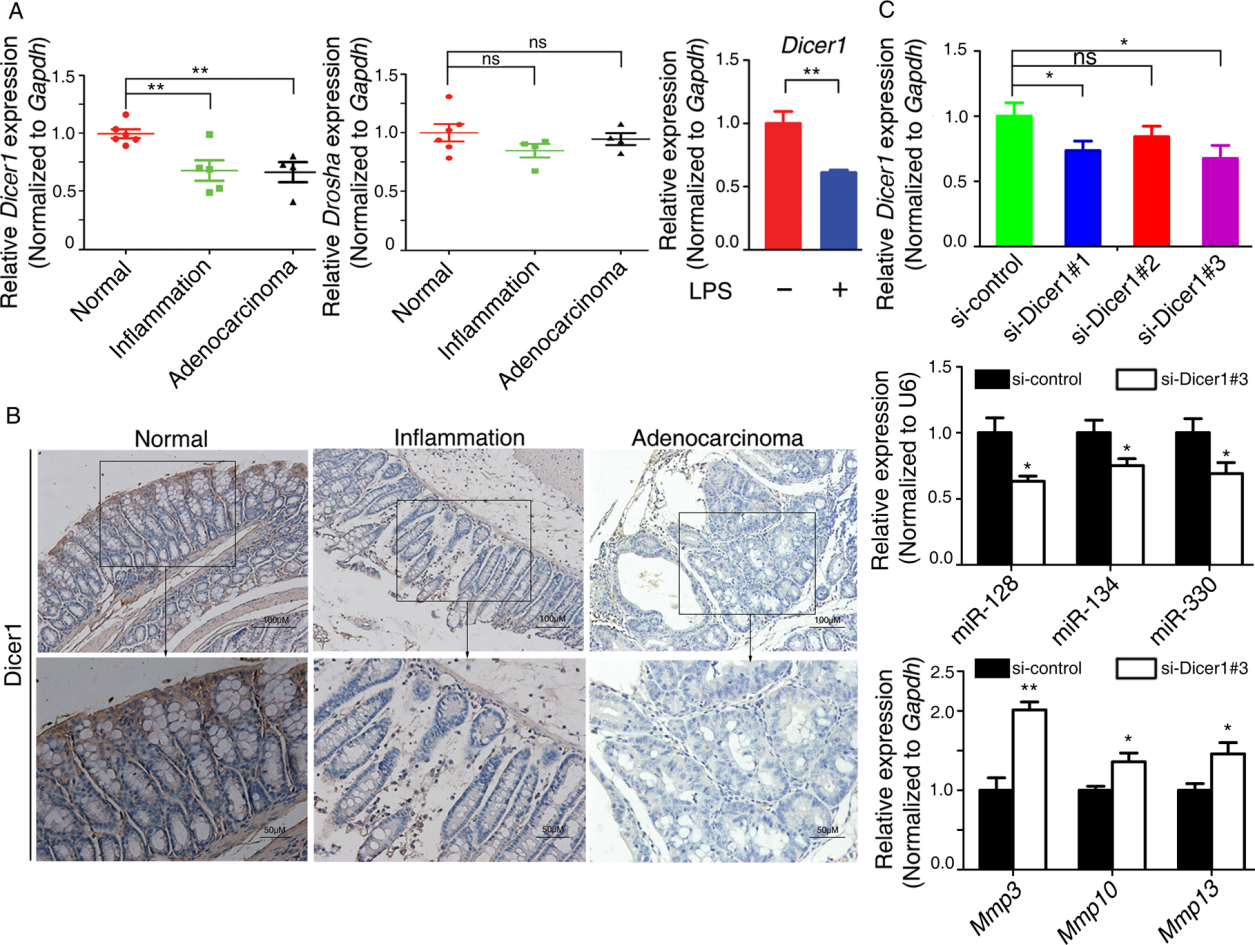
The mechanism responsible for the downregulation of miR-128, miR-134, and miR-330 during CAC progression **(A)** Left: qRT-PCR analysis of the mRNA levels of *Dicer1* and *Drosha* in mouse colitis tissue, CAC tissue, and normal colon tissue. Right: 6 h of LPS stimulation (100 ng/ml) of murine RAW264.7 macrophages decreased *Dicer1* mRNA expression as assessed by qRT-PCR. **(B)** Immunohistochemistry analysis of Dicer1 in mouse colitis tissue, CAC tissue, and normal colon tissue. **(C)** (Upper) Effects of three mouse Dicer1 siRNAs on Dicer1 mRNA expression in CT26.WT cells by qRT-PCR. (Middle) The mRNA levels of miR-128, miR-134, and miR-330 from CT26.WT cells were downregulated by siRNA targeting Dicer1. (Bottom) The mRNA levels of *Mmp3*, *Mmp10*, and *Mmp13* from CT26.WT cells were upregulated by siRNA targing Dicer1. All data are shown as the mean ± SD. *p < 0.05, **p < 0.01 compared with control.

Dicer1 and Drosha are key regulators of miRNA biogenesis [17]. We thus assessed the intracellular levels of Dicer1 and Drosha in RAW264.7 macrophages to verify whether their dysregulation played a role in the observed decrease in miR-128, miR-134, and miR-330 levels during CAC progression. In the AOM-DSS mouse model, *Dicer1* mRNA level was downregulated in colitis tissue and CAC tissue compared with normal colon tissue; the observed decrease in *Drosha* mRNA level was not significant (Figure 4A). The decrease in *Dicer1* mRNA level also was observed upon LPS stimulation (6 h) of RAW264.7 cells (Figure 4A). Immunohistochemistry also revealed that Dicer1 was downregulated in colitis tissue and CAC tissue compared with normal tissue (Figure 4B). Then we asked whether down-regulation of Dicer1 led to down-regulation of miR-128, miR-134 and miRNA-330, and consequently up-regulation of Mmp3/Mmp10/Mmp13 particularly? We addressed this question by transfecting CT26.WT murine colon cancer cells with three different siRNAs targeting Dicer1, respectively. qRT-PCR confirmed that the expression level of *Dicer1* was reduced in CT26.WT cells upon transfection with Dicer1 siRNAs, and #3 siRNA-Dicer1 is the most efficient one (Figure 4C). We found that knockdown of Dicer1 expression resulted in downregulation of miR-128/miR-134/miRNA-330 and upregulation of Mmp3/Mmp10/Mmp13 (Figure 4C). These results indicated Dicer1 was decreased in the inflammation–cancer link, and might contribute to the observed inhibition of miRNA biogenesis and increase of Mmps biogenesis during CAC progression.

### MiR-128, miR-134, and miR-330 suppress the tumorigenicity of murine colon cancer cells *in vitro* and *in vivo*

**Figure 5:**
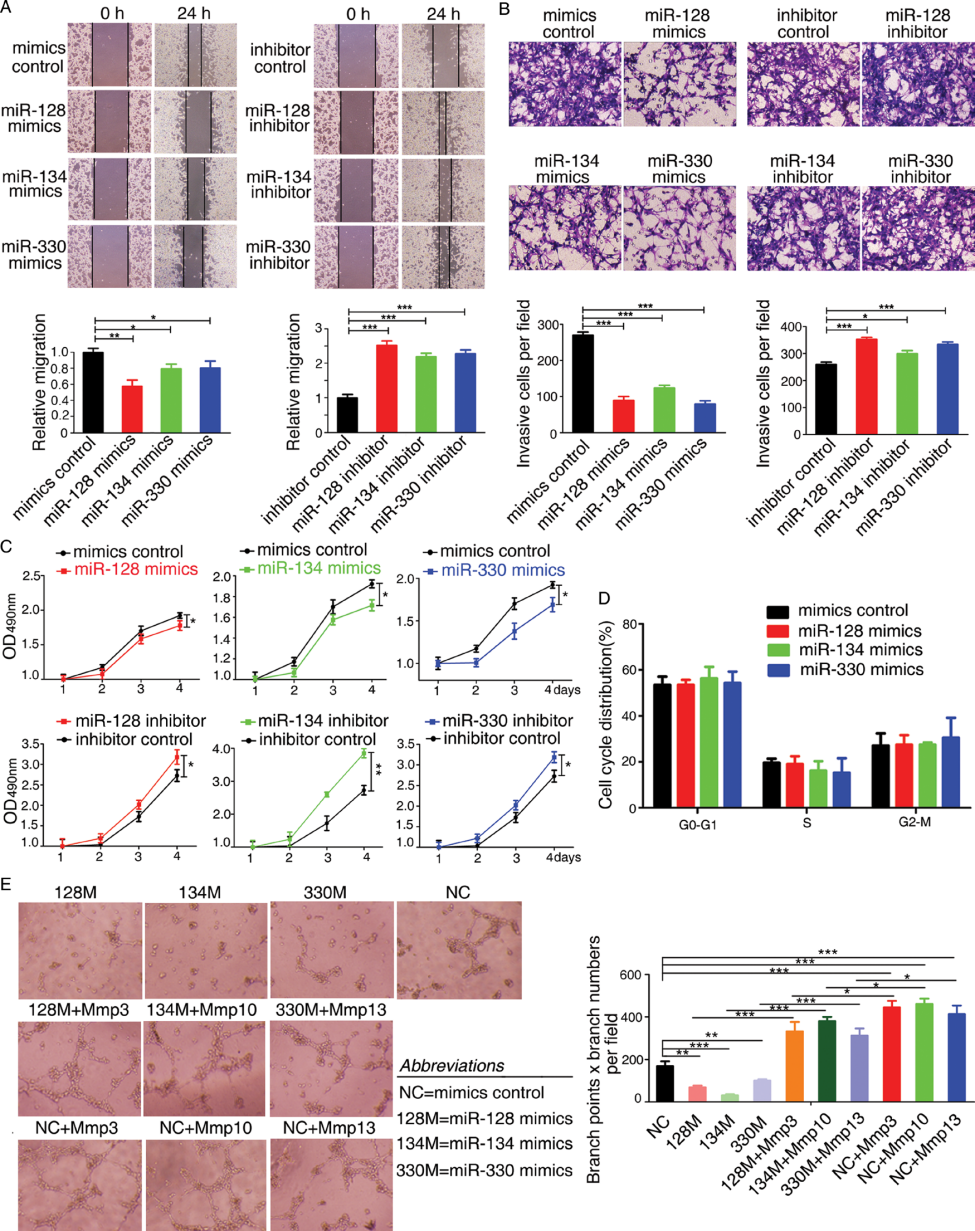
miR-128, miR-134, and miR-330 suppress the tumorigenicity of murine colon cancer cells and inhibit tube formation of endothelial cells *in vitro* **(A)** Wound-healing assay showing that miR-128, miR-134, and miR-330 suppressed the migration of CT26.WT cells. **(B)** Transwell migration assay showing that miR-128, miR-134, and miR-330 decreased the invasion ability of CT26.WT cells. Representative figures of the migrated stained cells are shown. The cells in five randomly selected areas were counted. The data are shown as the mean ± SD. **(C)** Ectopic expression of miR-128, miR-134, and miR-330 inhibits the proliferation of murine colon cancer cells. CT26.WT cells were plated in 96-well plates after transfection with miRNA-specific mimics, miRNA inhibitors, mimics control, or inhibitor control. Cell proliferation was assessed on the indicated days using the MTT assay. **(D)** Flow cytometry analysis of the cell cycle distribution of CT26.WT cells transfected with the miR-128, miR-134, and miR-330 mimics or mimics control. Bar graph indicates the percentage of cells remaining in phases G_0_–G_1_, S, and G_2_. **(E)** Tube formation of HUVECs was suppressed by treatment with medium preconditioned with CT26.WT cells overexpressing miR-128, miR-134, or miR-330 when compared with that of the control group cells. Transfection of cells with *Mmp3*, *Mmp10*, or *Mmp13* rescued the angiogenic capabilities of cells overexpressing miR-128, miR-134, or miR-330. All data are shown as the mean ± SD. *p < 0.05, **p < 0.01, ***p < 0.001 compared with control.

Since miR-128, miR-134, and miR-330 can target Mmp3, Mmp10, and Mmp13, respectively and are downregulated in the inflammation-cancer link, we next explored the functions of miR-128, miR-134, and miR-330 with respect to their contributions to the tumorigenic potential of the CT26.WT murine colon cancer cells, including their effects on the cell cycle and cell proliferation, migration, and invasion. The functions of these miRNAs were assessed by transfecting the cells with miR-128, miR-134, and miR-330 mimics (or the corresponding chemically synthesized miRNA inhibitors). The wound-healing assay showed that all three miRNAs disrupted cell migration. Overexpression of the miR-128, miR-134, or miR-330 mimic clearly delayed wound gap closure compared with each mimic control, whereas knockdown of miR-128, miR-134, or miR-330 using the corresponding miRNA inhibitor had the opposite effect (Figure 5A). The matrigel invasion assay showed that overexpression of the miR-128, miR-134, or miR-330 mimic inhibited the *in vitro* invasive potential of CT26.WT cells, whereas knockdown of the miRNAs promoted the invasive potential (Figure 5B presents representative images of the migrated/stained cells). We next used the methyl thiazolyl tetrazolium (MTT) assay to assess the effect of each miR-128, miR-134, and miR-330 mimic on cell proliferation. As shown in Figure 5C, overexpression of the miR-128, miR-134, or miR-330 mimic attenuated CT26.WT cell proliferation, and knockdown of each miRNA had the opposite effect. Cell cycle analysis using flow cytometry revealed that the three miRNA mimics did not affect the cell cycle distribution of CT26.WT cells (Figure 5D). We next explored whether the miR-128, miR-134, and miR-330 mimics could affect angiogenesis using an endothelial cell tube formation assay. As shown in Figure 5E, medium from CT26.WT cells overexpressing miR-128, miR-134, or miR-330 inhibited tube formation. Transfection with a construct encoding *Mmp3*, *Mmp10*, or *Mmp13* rescued the angiogenic potential of the cells overexpressing each of the miRNAs. These observations suggested that miR-128, miR-134, and miR-330 can suppress the migration, invasion, and proliferation of murine colon cancer cells *in vitro* and that they also can inhibit endothelial cell tube formation to some extent.

**Figure 6:**
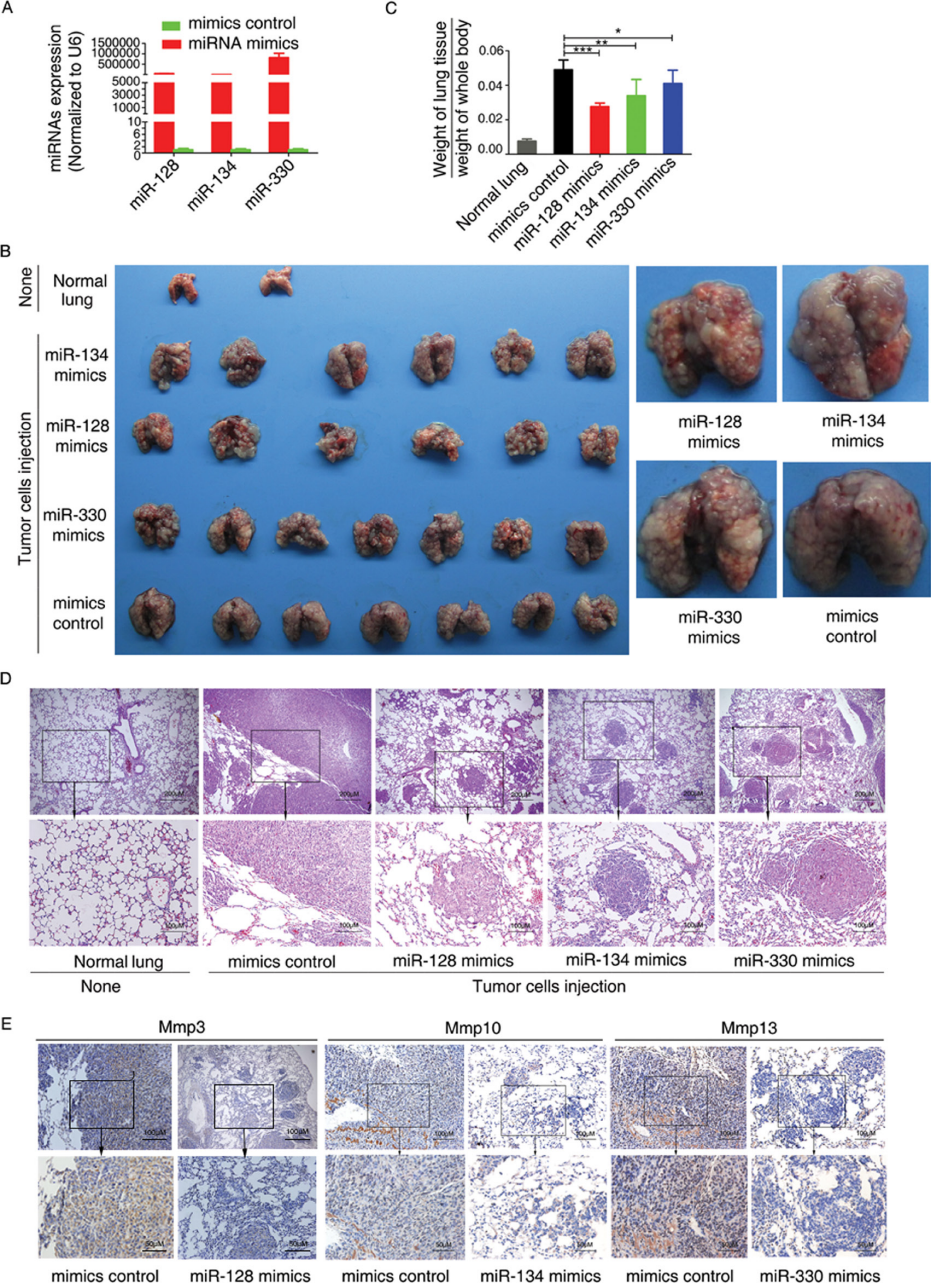
miR-128, miR-134, and miR-330 suppress the metastasis of murine colon cancer cells in a nude mouse xenograft model **(A)** Effects of transfection with miRNA mimics on miRNA expression in CT26.WT cells. **(B)** The tail vein of nude mice was intravenously injected with murine colon cancer cells (CT26.WT) transfected with miR-128, miR-134, or miR-330 mimics or mimics control. After 14 days, the mice were sacrificed, necropsies were performed, and images of whole lung tissue were acquired. **(C)** The weight of the nude mice was measured every 3 days. After sacrifice, lung tissue was excised and weighed, and the ratio of (weight of lung tissue)/(weight of whole body) was calculated. **(D)** Representative images of hematoxylin and eosin–stained lung sections, which revealed the metastatic nodules. **(E)** Immunohistochemical staining revealed that transfection of CT26.WT cells with miR-128, miR-134, or miR-330 mimics resulted in decreased expression of Mmp3, Mmp10, or Mmp13, respectively, within the resulting tumors. All data are shown as the mean ± SD. *p < 0.05, **p < 0.01, ***p < 0.001 compared with control.

Because overexpression of miR-128, miR-134, or miR-330 could inhibit tumorigenesis *in vitro*, we next asked whether these miRNAs could inhibit the metastatic potential of CT26.WT cells *in vivo*. Cells that had been transfected with an miR-128, miR-134, or miR-330 mimic or control mimic were injected into the tail vein of nude mice, and the efficiency of transfection was verified (Figure 6A). Lung metastasis was then quantified either by determining mean total lung weight of 6–7 lungs for each group or by serial sectioning to determine the mean number of metastases per lung [18]. Nude mice injected with the mimic control cells succumbed to massive lung metastases (Figure 6B), whereas mice that received cells transfected with the miR-128/−134/−330 mimics had relatively decreased lung weight and decreased ratio of lung weight to whole body weight compared with the control group (Figure 6C). The lungs of mice that received miRNA (−128/−134/−330) mimic–transfected cells were significantly smaller and lighter compared with the mimics controls, which suggested smaller lung lesions. Moreover, nude mice injected with cells overexpressing miR-128, miR-134, or miR-330 mimic had significantly fewer macroscopic lung metastases compared with the mimic controls (Figure 6D). Further, immunohistochemical staining revealed that transfection with the miR-128, miR-134, or miR-330 mimic resulted in decreased expression of Mmp3, Mmp10, or Mmp13, respectively, within tumors (Figure 6E). These results indicated that miR-128, miR-134, and miR-330 can suppress the metastasis of murine colon cancer cells to lung. Overall, the results demonstrate that these three miRNAs counter the malignant phenotype of colon cancer both *in vivo* and *in vitro*.

## DISCUSSION

The prevalence of cancer related to chronic infection has reached approximately 1.9 million cases per year, or 17.8% of the global cancer burden [19]. It is generally believed that chronic inflammation plays a prominent role in tumorigenesis [2]. Different aspects of inflammation appear to regulate different stages of carcinogenesis—from cancer initiation to invasion and metastasis and even morbidity and mortality [20]. Ulcerative colitis is a worldwide healthcare problem of continuously increasing incidence, characterized by chronic relapse of intestinal inflammation and strong association with colon cancer [5]. Our previous findings found that peritoneal M2 macrophages played a critical role in ulcerative colitis associated carcinogenesis, including unbalanced proinflammatory and anti-inflammatory axis and enhanced expression of migration/invasion-associated factors [21]. Popivanova et al. reported that blockade of a chemokine, CCL2, can significantly reduce chronic colitis-associated carcinogenesis in mice [22]. Although the genetic, environmental, and microbial factors and the immune responses that underlie the pathogenesis have been identified, the etiology of ulcerative colitis remains largely unknown. In this regard, more effort must be made to identify not only the relevant genes but also the specific mechanisms by which those genes are regulated and how **t**heir altered regulation contributes to disease.

The AOM/DSS-induced CAC mouse model has proven useful for exploring the pathogenesis and assessing the therapeutic effect of drugs. Based on our previous observations [7], we noted that the expression of *Mmp3*, *Mmp10*, and *Mmp13* was dramatically increased from inflammation to cancer, particularly during acute inflammatory stage (~200-fold increase; Figure 1A). In accordance with such findings, we found the same phenomenon which *Mmp3*, *Mmp10*, and *Mmp13* were upregulated in acute LPS stimulation of RAW264.7. Mmps have important physiological functions and are closely associated with tumor formation and invasive capacity, acting as crucial molecular targets. Kun Shang *et al*. reported that neutrophils induced colon carcinogenesis through tumor-associated angiogenesis by MMP-9 and cell proliferation by neutrophil elastase. They also found MMP-9 mRNA expression was remarkably enhanced during murine CAC progression [23]. Moreover, it has been reported that MMPs, especially MMP-7 and MMP-13, which are expressed primarily on the tumor cell surface, are elevated in inflammatory bowel disease, which may have more chance to evolve into malignancy than normal tissue [24]. A large number of clinical observations suggested that the prognosis of disease may have been established in its initial stage and early treatment often can get better effects. Thus reducing the expression of MMPs in the early stage may benefit in the patients with ulcerative colitis.

The regulatory mechanisms underlying the expression of Mmps primarily manifest at the levels of transcription, zymogen activation, and targeted inhibition. As evidenced by the first demonstration of miRNA actions in *Caenorhabditis elegans*, including that of let-7 [25, 26], an increasing body of evidence indicates that miRNAs play critical roles in regulating gene expression. For example, miR-155 [12, 27], miR-98 [13], miR-210 [28], miR-146 [29], miR-34a [30] have been reported to participate in the production of proinflammatory cytokines and in targeting key molecules of the innate immune response. These established roles of miRNAs in various inflammatory conditions suggested that miRNAs may in fact target Mmp mRNAs during inflammation. In the present study, we identified miR-128, miR-134, and miR-330 as negative regulators of *Mmp3*, *Mmp10*, and *Mmp13*, respectively. MiR-128, miR-134, and miR-330 have been reported to play substantive roles in regulating cell proliferation, survival, motility, apoptosis, and invasion [31–35]. Colitis-associated cancer and sporadic colorectal cancer had different pathogenesis, but they had the same properties as a malignant tumor. Thus we tested the effect of miR-128, miR-134, and miR-330 on the metastasis-related aspects in murine colon cancer cells. MiR-128, miR-134, and miR-330 overexpression in murine colon cancer cells attenuated the ability of the cells to proliferate, migrate, and invade other tissues. The experiment with nude mice further revealed that these three miRNAs could suppress pulmonary metastasis of murine colon cancer cells.

We investigated the mechanisms by which the levels of these three miRNAs are regulated. Dicer1 and Drosha serve as key regulators of miRNA biogenesis, and alterations in their intracellular levels may contribute to widespread miRNA deregulation in cancers [17]. Downregulation or lower expression of DICER1 is significantly associated with lung cancer survival [36], breast and endometrial cancer progression and recurrence [37], or stemness and metastatic characters of colon cancer [38]. Tarallo *et al.* reported that DICER1 deficiency activates the NLRP3 inflammasome and triggers Toll-like receptor–independent MyD88 signaling via IL-18 in the retinal pigmented epithelium [39]. To verify whether dysregulation of Dicer1 and Drosha participate in downregulation of miRNAs during CAC progression, we assayed the expression levels of Dicer1 and Drosha in AOM/DSS-induced colitis and in CAC mice and found that Dicer1 level was decreased during CAC progression. A macrophage model of inflammation also verified this result. Furthermore, we indicated that knockdown of Dicer1 resulted in downregulation of miR-128/miR-134/miRNA-330 and upregulation of Mmp3/Mmp10/Mmp13 (Figure 4C). Thus, the downregulation of Dicer1 may partly explain the mechanism of miRNA downregulation and Mmps upregulation during CAC progression.

In conclusion, we report that Mmps are key molecules in non-resolving inflammation, and their expression levels are regulated, at least part, by miRNAs. The expression levels of these miRNAs are influenced by Dicer1. Our study also identifies new functions for these miRNAs. Understanding the key molecules involved in non-resolving inflammation and their mechanism of regulation, especially that of the miRNA pathway, will provide additional opportunities for the design of treatments for CAC.

## MATERIALS AND METHODS

### Patient tissue specimens

A total of 5 colorectal cancer tissues and 5 adjacent normal colorectal tissues were obtained from The Third Xiangya Hospital of Central South University in 2014. The adjacent normal tissue samples were obtained from the normal colorectal tissue located 5 cm away from the tumor. All cases were confirmed by pathologists. The patients received neither chemotherapy nor radiotherapy before surgery. Written informed consents were obtained from patients and with approval for experiments from the ethical review committees of the appropriate institution.

### Cell lines and culture

The murine colon cancer cell line CT26.WT, murine macrophages (RAW264.7), and human umbilical vein endothelial cells (HUVECs) were cultured in RPMI-1640 medium supplemented with 10% fetal calf serum. HEK293 cells were cultured in Dulbecco's modified Eagle's medium (Invitrogen, Carlsbad, CA, USA) with 1 g/l glucose and 10% fetal calf serum.

### Bioinformatics

Potential murine miRNA target sites of *Mmp3*, *Mmp10*, and *Mmp13* mRNAs were predicted and analyzed using four publicly available bioinformatics websites: TargetScan, miRWalk, microRNA.org, and RNA22. We integrated the results from the four programs and selected several common murine miRNAs that putatively target Mmps for further biochemical validation. We utilized DAVID Bioinformatics Resources 6.7 Analysis Tools (http://david.abcc.ncifcrf.gov/) to classify the function of the target genes, which were predicted from TargetScan, miRWalk, and miRanda.

### Constructs

The 3′-UTR of each of *Mmp3* and *Mmp10* mRNA was amplified and subcloned into pmir-Report luciferase vector (Ambion, Austin, TX, USA). Mutants of the seed region of the putative miRNA-binding sites in each 3′-UTR of each mRNA (LR-128/*Mmp3* m1, LR-128/*Mmp3* m2, LR-134/*Mmp10* m) were generated by point mutation PCR. All primers used are described in Supplemental Table 1. With respect to *Mmp13*, we directly synthesized sense and antisense strands of its 3′-UTR that contained the binding site for miRNA-330. The sense and antisense oligonucleotide strands were annealed, digested with Hind*III* and Spe*I*, and ligated into pmir-Report. Then two single strands representing the 3′-UTR of *Mmp13* harboring changes in the bases within the binding site for miRNAs were synthesized as a mutant control. The full-length coding sequences of murine Mmp3, Mmp10, and Mmp13 were subcloned into the expression vector pIRES (Clontech, Mountain View, CA, USA). The oligonucleotides used in these studies are also shown in Supplementary Table 1.

### Oligonucleotides and transfection

Synthetic miRNA mimics, 2′-O-methyl antisense oligonucleotides targeted toward miRNAs (miRNA inhibitors), and negative controls (mimics control or inhibitor control) were purchased from GenePharma (Shanghai, China). CT26.WT cells in culture were trypsinized, counted, and seeded into 6-well plates. Transfection with the miRNA mimics or miRNA inhibitors was carried out using HiPerFect Transfection Reagent (Qiagen, Valencia, CA, USA). Three small interfering RNAs (siRNAs) (synthesized by Ribobio, Guangzhou, China) were used to target mouse Dicer1. A nonsilencing siRNA (si-control) was used as control. The si-RNA sequences are provided in Supplementary Table 2. At 48 h post-transfection, qRT-PCR was used to verify the transfection efficiency, and experiments were performed as described below.

### Quantitative real-time PCR

cDNA was synthesized from 2 μg of total RNA by means of a reverse transcription kit (Fermentas, Glen Burnie, MD, USA). Mouse *Gapdh* was amplified in parallel as an internal control. Expression of the mRNAs was evaluated using SYBR green qRT-PCR (TaKaRa, Otsu, Japan) in accordance with the standard protocol. The individual miRNA qRT-PCR Quantitation kit was purchased from Genepharma, and U6 small nuclear RNA was used as an endogenous control for assay of miRNA expression levels. Expression of each gene was quantified by measuring cycle threshold (Ct) values and normalized using the 2^−ΔΔCt^ method relative to U6 small nuclear RNA or *Gapdh* mRNA. The qRT-PCR primers are listed in Supplementary Table 3.

### Luciferase reporter assay

Luciferase activity was measured according to the dual-luciferase assay manual (Promega, Madison, WI, USA). The Renilla luciferase signal was normalized to the firefly luciferase signal for each individual construct in each analysis.

### Western blotting

Western blotting was carried out as described [40]. Antibodies against Mmp3 and Mmp13 were purchased from Abcam (Cambridge, MA, USA). Antibodies against Mmp10, Dicer1, and Gapdh were purchased from Millipore (Billerica, MA, USA). Antibody against Erk1 was from Santa Cruz Biotechnology (Santa Cruz, CA, USA).

### Immunohistochemistry

Immunohistochemistry was carried out as described [7]. The antibody against Mmp13 was purchased from Abzoom (Dallas, TX, USA). Antibodies against Mmp3, Mmp10, and Dicer1 were the same ones that were used for western blotting. Omission of the primary antibody was used as negative control.

### Wound-healing assay

CT26.WT cells (1 × 10^6^) were cultured overnight. The next day the cells were transfected with a construct encoding a synthetic miRNA mimic or inhibitor and then grown in a 6-well plate. After the cell monolayer had reached 90% confluency, a wound was made with a 10-μl pipette tip. Cells were then cultured in medium with 1% serum, and migration at the corresponding wound site was documented using a microscope at 0 and 24 h.

### Matrigel invasion assay

Before cell seeding, 24-well Transwell plates (8-μm pores; Corning, New York, NY, USA) were coated with Matrigel Matrix (BD Biosciences, San Diego, CA, USA). Transfected cells (1 × 10^5^) suspended in 200 μl volume of RMPI 1640 were added to the top of each insert well. Normal growth medium was placed in the bottom wells. The cells were then allowed to migrate for 24 h at 37°C. The emigrated cells were fixed with 10% methanol for 15 min and allowed to air dry at room temperature. Then the invasive cells on the lower surface of the membrane were stained with 2% crystal violet for 5 min, and the stained cells were counted under a microscope. To minimize bias, at least five and only selected fields with 100× magnification were counted, and the various counts were averaged.

### Tube formation assay

The tube formation assay was carried out as described [41]. In brief, 96-well plates were coated with 50 μl Matrigel. HUVECs were suspended in RPMI-1640 medium preconditioned with CT26.WT cells. HUVECs (20 000) were added to each well and incubated at 37°C overnight. The CT26.WT cells were previously cotransfected with an individual miRNA mimic and an expression vector for a Mmp coding sequence for 48 h. Images were acquired under an inverted microscope. Antiangiogenic activity was quantified by measuring the length of tube walls formed between discrete endothelial cells in each well relative to the control.

### Cell cycle analysis

At 48 h post-transfection with the individual miRNA mimics (40 nM), CT26.WT cells were collected and washed with precooled phosphate-buffered saline. Cells were fixed with 70% ethanol and stored at 4°C with rotation overnight. On the following day, fixed cells were washed with precooled phosphate-buffered saline and stained with propidium iodide (50 μg/ml) for 30 min at 37°C in the dark. The stained cells were analyzed with a MoFlox XDP flow cytometer (Beckman Coulter, Fullerton, CA, USA). At least 10 000 cells in each sample were analyzed to obtain a quantifiable averaged signal.

### MTT assay

After the appropriate transfection, CT26.WT cells were seeded at a density of 2 × 10^3^ cells/well in 200 μl in 96-well plates. On days 1–4, 20 μl MTT solution (5 mg/ml) was added to each well. The plate was incubated at 37°C for an additional 4 h, the medium was removed, and 150 μl dimethyl sulfoxide was added to each well. The plates were shaken for 10 min to dissolve the MTT formazan crystals. The optical density at 490 nm of each well was measured with a scanning multiwell spectrophotometer. The experiment was repeated three times, and six parallel samples were measured each time.

### Mouse models and experiments with nude mice

The AOM/DSS-induced colitis mouse model was created as described by Tang *et al* [7]. Four-week-old male BALB/c nude mice were used to examine tumor metastasis. To assess the effect of each miRNA (miR-128, miR-134, miR-330) on tumor metastasis, CT26.WT cells that had been transfected with miR-128, miR-134, and miR-330 mimics (or mimics control) were injected into the tail vein of nude mice. After 14 days, necropsies were performed. The micrometastases in lung per section in individual mice were counted by morphological observation and hematoxylin and eosin staining. The experiments were performed using six to seven mice per group (for different miRNA mimics), and all animal procedures were performed in accordance with institutional guidelines.

### Statistical analysis

The significance of differences between groups was assessed using the Student's *t* test in SPSS 17.0 and GraphPad Prism 5. Correlation analysis was performed using the Spearman method in SPSS 17.0. A *p*-value of < 0.05 was considered to be statistically significant.

## SUPPLEMENTARY FIGURE AND TABLES


