# Supplementary material for: Up-regulation of matrix metalloproteinases in a mouse model of chemically induced colitis-associated cancer: the role of microRNAs
Source: Oncotarget. 2015 Jan 23;6(7):5412–25. doi: 10.18632/oncotarget.3027 (PMC4467157; doi:10.18632/oncotarget.3027)
Supplement: Supplementary file 1 [file oncotarget-06-5412-s001.pdf]

SUPPLEMENTARY FIGURE AND TABLES

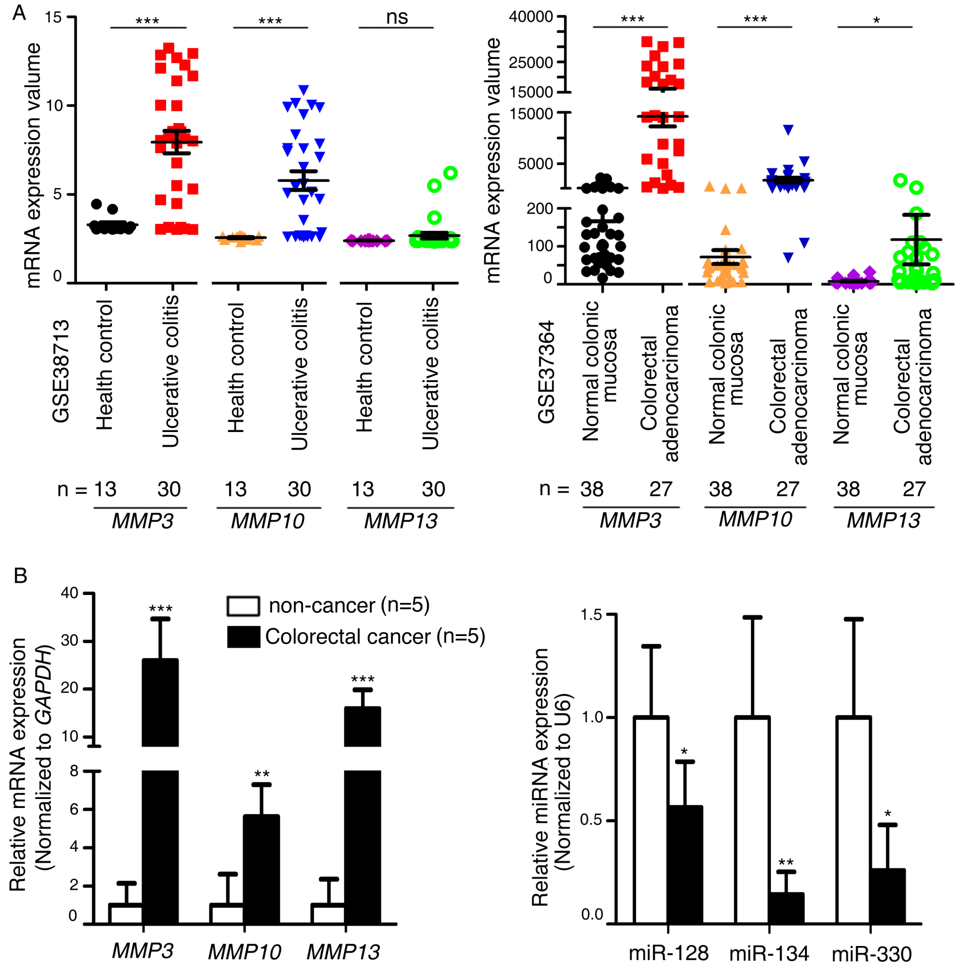

Supplementary Figure 1: The expression levels of *Mmp3*, *Mmp10*, and *Mmp13* correlated negatively with those of miR-128, miR-134, and miR-330, respectively, in human colorectal cancer specimens. (A) mRNA expression of *Mmp3*, *Mmp10*, and *Mmp13* in human normal, ulcerative colitis and colorectal adenocarcinoma tissues as obtained from NCBI gene expression profile database (#GSE38713, #GSE37364). **(B)** Expression levels of *Mmp3/10/13* (left) and miR-128/-134/-330 (right), as assessed by qRT-PCR in 5 human colorectal tumor and adjacent normal tissues.

Supplementary Table 1: Primer and oligonucleotides list for constructs

3′UTR of *Mmp3*	Forward	5′-GTGACGCGTAAAGAGATCCAAGGAA-3′
	Reverse	5′-GCGAAGCTTTTAATAAACGACACAC-3′
3′UTR of mutant1 *Mmp3*	Forward	5′-CAAAGGATGTTCAGAAGGGGAAAATAGCTTACACTGTGTCC-3′
	Reverse	5′-GGACACAGTGTAAGCTATTTTCCCCTTCTGAACATCCTTTG-3′
3′UTR of mutant2 *Mmp3*	Forward	5′-AGCACTGTGTAGCTTAAAAAAAATCCCAAGGAGAGGAG-3′
	Reverse	5′-CTCCTCTCCTTGGGATTTTTTTTAAGCTACACAGTGCT-3′
3′UTR of *Mmp10*	Forward	5′-GGCACGCGTTCATCATGACAAGACA-3′
	Reverse	5′-GCGAAGCTTTTCAAAAATAAAGCTC-3′
3′UTR of mutant *Mmp10*	Forward	5′-CAGATGTCTTTCATAATGCCCTTTTAAGCATCACCTGAGCA-3′
	Reverse	5′-TGCTCAGGTGATGCTTAAAAGGGCATTATGAAAGACATCTG-3′
3′UTR of *Mmp13*	Forward	5′-CGCGTAAGTTGTTATTTATCTCCCAGAGAGTATTTGGAATACTTTCAGAT GTATGA-3′
	Reverse	5′-AGCTTCATACATCTGAAAGTATTCCAAATACTCTCTGGGAGATAAATAA CAACTTA-3′
3′UTR of mutant *Mmp13*	Forward	5′-CGCGTAAGTTGTTATTTATCTTTTCCCCCGTATTTGGAATACTTTCAGA TGTATGA-3′
	Reverse	5′-AGCTTCATACATCTGAAAGTATTCCAAATACGGGGGAAAAGATAAATA ACAACTTA-3′
CDS of *Mmp3*	Forward	5′-ATCGATATCATGAAAATGAAGGGTC-3′
	Reverse	5′-TATGGATCCTTAACAATTAAACCA-3′
CDS of *Mmp10*	Forward	5′-ATCGCTAGCATGGAGCCACTAGCC-3′
	Reverse	5′-TATGAATTCTCAGCACAGCAGCCAG-3′
CDS of *Mmp13*	Forward	5′-ATCGCTAGCATGCATTCAGCTATC-3′
	Reverse	5′-TATGGATCCTTAACACCACAATATG-3′

Supplementary Table 2: Sequences of siRNA

**Si-RNA**		**sequences**
Si-Dicer1#1	Forward	5′-GCGCAAAUACAAGCCCUAU dTdT-3′
	Reverse	5′-AUAGGGCUUGUAUUUGCGC TdTd-3′
Si-Dicer1#2	Forward	5′-GCCGAUCUCUAAUUACGUA dTdT-3′
	Reverse	5′-UACGUAAUUAGAGAUCGGC TdTd-3′
Si-Dicer1#3	Forward	5′-GCAUGCUAUCACCACAUAU dTdT-3′
	Reverse	5′-AUAUGUGGUGAUAGCAUGC TdTd-3′

Supplementary Table 3: Primer list for real-time PCR

Gene	Forward	Reverse
Mmp3-mouse	5′-CAGACTTGTCCCGTTTCCAT-3′	5′-GGTGCTGACTGCATCAAAGA-3′
Mmp10-mouse	5′-CCTGCTTTGTCCTTTGATTCAGT-3′	5′-CGGGATTCCAATGGGATCT-3′
Mmp13-mouse	5′-TGATGAAACCTGGACAAGCA-3′	5′-TCCTCGGAGACTGGTAATGG-3′
Dicer1-mouse	5′-TCGAGCCTCCATTGTTGGTC-3′	5′-CGGGTTTGGGGTAACTCTCC-3′
Gapdh-mouse	5′-TCTGACGTGCCGCCTGGAGA-3′	5′-CAGCCCCGGCATCGAAGGTG-3′
MMP3-human	5′-CGAGTCACACTCAAGGGAACT-3′	5′-CGAGTGCTTCCCCTTCTCTT-3′
MMP10-human	5′-TTTGACCCCAATGCCAGGAT-3′	5′-GCAAGGCTCATCTTCTTCAGTC-3′
MMP13-human	5′-GCCATTACCAGTCTCCGAGG-3′	5′-TACGGTTGGGAAGTTCTGGC-3′
GAPDH-human	5′-AACGGATTTGGTCGTATTGG-3′	5′-TTGATTTTGGAGGGATCTCG-3′
